# The Message or the Messenger: The Impact of Explanation Satisfaction and Source Characteristics on Perceptions of Experts and Verdicts

**DOI:** 10.3390/bs15121670

**Published:** 2025-12-03

**Authors:** Kristen L. Gittings, Carly E. Giffin, Jessica M. Salerno

**Affiliations:** 1 Department of Psychology, Cornell University, Ithaca, NY 14850, USA; 2 Federal Judicial Center, Washington, DC 20002, USA

**Keywords:** expert witnesses, explanation satisfaction, gender, education, expertise

## Abstract

Understanding how expert characteristics shape verdicts is critical. This study identified message (e.g., explanation satisfaction) and source characteristics (e.g., education) that predict perceived expertise and verdicts. We hypothesized an expert with more (versus less) satisfying explanations, education, and hands-on contact with the evidence would be perceived as higher in expertise, and that female experts would be perceived as lower in expertise than male experts. Further, higher expertise would predict more verdicts in line with the expert’s testimony. In three mock jury studies (N = 1853), we measured (Studies 1–3) and manipulated (Study 3) explanation satisfaction. We also manipulated source characteristics (i.e., education, Studies 1–3; evidence contact, Studies 1–2; expert gender, Studies 2–3). Mock jurors reported their verdicts and perceptions of the expert. Satisfaction with the expert’s explanation was the only consistent predictor of perceived expertise, and in turn, verdicts. This effect operated independently from source characteristics; expert gender, education, and more hands-on contact with the evidence did not have consistent influence on satisfaction. We found evidence that expertise cues (education, Studies 1–3; evidence contact, Studies 1–2) impacted explanation satisfaction but did not consistently influence verdicts. Our findings suggest that explanation satisfaction—not source characteristics—reliably predicted expertise and verdicts. Thus, attorneys should ensure the experts they retain can effectively communicate to jurors.

## 1. Introduction

For decades, courts have turned to the cutting-edge science of the day to help them address legal issues. Although the use of scientific evidence in a trial is not new, scientific evidence remains an ever-increasing and evolving part of our legal system. Further, as science continues to advance at a rapid pace, it is becoming more and more difficult for non-scientists to understand and evaluate it—a critical role of legal factfinders. Indeed, jurors and judges have difficulty differentiating sound from methodologically flawed science (McAuliff et al., 2009; Schweitzer & Saks, 2011; Krauss & Sales, 2001). Trials are in need not only of the most accurate scientific evidence available, but also of effective experts who can clearly, accurately, and compellingly explain science to judges and juries.

Federal Rule of Evidence 702 states that a person qualified as an expert by “knowledge, skill, experience, training, or education” can be introduced when he or she “will assist the trier of fact to understand the evidence or to determine a fact in issue” (Federal Rules of Evidence, 2019). The Federal Rules and courts across the country allow for a variety of people from a variety of backgrounds to serve as experts, depending on the kind of expertise a case may need. Yet, bias favoring some experts more than others based on their background remains (e.g., experts with more extensive experience, Koehler et al., 2016; experts with degrees from a prestigious university, Cooper et al., 1996). This suggests that in order to be effective, an expert may need to combat preconceived notions held by the jurors about what an expert *should* look like or what kind of experience and education an expert *should* have. Experts who do not reflect these expectations may be less convincing to jurors despite their objective expertise in the domain. The current study expands the expert witness literature by independently testing the impact of the often-conflated source factors of an expert’s education and experience with the evidence and whether the impact of those factors depends on the expert’s gender. Further, we also explore whether a juror’s satisfaction with an expert’s explanation, a subjective characteristic of their message, impacts the perceived expertise and influence of the expert on verdicts.

### 1.1. The Impact of Source Characteristics on Evaluations of Experts

The characteristics of an expert, often referred to as “source characteristics,” can impact how laypeople perceive an expert (e.g., how knowledgeable they are) and the degree to which jurors integrate expert testimony into their verdict decisions. Source factors can include things like prestige of education (e.g., Cooper et al., 1996; Koehler et al., 2016), assuming bias based on the expert being hired by one party too often (i.e., being a “hired gun”, Cooper & Neuhaus, 2000), and the field the expert is representing (e.g., Saks & Wissler, 1984). For jurors, consideration of source characteristics is not necessarily irrational or irrelevant. For instance, which side hired a witness is relevant to a careful evaluation of their testimony and any preconceived biases the expert may hold. However, some characteristics of the expert (e.g., expert gender) should have no bearing on evaluations of their testimony. Therefore, it is important to investigate which source factors may influence jurors’ evaluations of the testimony and legal decisions.

#### 1.1.1. Expertise Cues

A meta-analysis of persuasion studies found that the source factor with the most influence on judgments is the speaker’s perceived “expertise” (Wilson & Sherrell, 1993). In both research and the courtroom, expertise can span context, disciplines, and roles. Further, different studies use different criteria for what constitutes expertise (e.g., college degree, years of experience; Wilson & Sherrell, 1993). Some studies may be unintentionally conflating several different source factors and referring to all of them as “expertise.”

One way that researchers frequently manipulate expertise is through education: a witness with more education is considered more of an expert than one who has less education. Education is undoubtedly an important aspect of expertise, but participants may unconsciously infer other aspects of expertise when they are told about a speaker’s degree. That is, it would be reasonable for a participant to hold a heuristic that a speaker with a PhD might also have a more prestigious job, more work experience, and be older than a witness without a PhD. Thus, the expertise manipulation may have unintentionally manipulated additional source characteristics of job prestige, years of experience, and age along with education. In some experiments, authors intentionally varied several different factors to increase perceived expertise simultaneously (e.g., Koehler et al., 2016), sensibly believing that having one witness who is older, more educated, and more experienced than another witness would be a strong manipulation of expertise. These kinds of manipulations, while entirely reasonable, leave open the question of which aspect(s) were independently influential. The Federal Rules of Evidence note that an expert could be considered an expert based on their “knowledge, skill, experience, training, or education” (Federal Rules of Evidence, 2019). These are considered *separate* ways that a person could be qualified as an expert. A mechanic might be qualified as an expert based on their years of experience servicing cars, despite a lack of any formal education. Conversely, a psychologist might be qualified as an expert based on their education, even if they have never personally interviewed a defendant in a case. Many previous studies do not consider this separation, leaving us with the question of which aspect of expertise affected jurors’ perceptions of expertise and case judgments in the past studies.

The link between education and expertise seems relatively clear—more of the former suggests more of the latter. The link between the extent and nature of contact with the evidence and expertise, however, could use clarification. Perhaps experts who are physically handling evidence (e.g., by collecting it from the crime scene or analyzing it in the lab) may be seen as more credible than those who read the reports prepared by others, interpret these findings, and explain them to the public. This idea has roots in recent work on laypeople’s attitudes toward science. An increase in the general public’s lack of trust in scientists, scientific findings, and even higher education in general has been documented in the media (e.g., Kennedy & Tyson, 2023), as well as in the very research they may distrust (e.g., Gauchat, 2012; Rutjens et al., 2018). It is possible, then, that some of the legal misunderstandings of scientific evidence might not be a lack of understanding but rather a rejection of scientists whom they perceive to be aloof and out of touch. If this is the case, then someone who actually performs the task—collecting and processing the evidence—may be seen as having more expertise and, as a result, might exert more influence over verdicts than a supervisor or hired expert who has merely reviewed a report and shown up to court—even if they have impressive education credentials. Thus, even if this person has less education and a less prestigious title, their hands-on experience or proximity to the evidence being evaluated may make them a more persuasive expert to jurors.

Studying the independent impact of these cues for expert witnesses is particularly vital given that perceptions of expertise differ based on context. Jurors place more value on factors that signal an expert witness’s “expertise” when faced with situations where information is incomplete (Maddux & Rogers, 1980), the task includes uncertainty (Chaiken & Maheswaran, 1994; Melamed & Savage, 2016), or if high-quality evidence is presented (Flick et al., 2022). The current studies attempt to disentangle the independent effects of an expert’s education and degree of direct contact with the evidence on perceived expertise and mock jurors’ satisfaction with the testimony while holding all other aspects of the expert and testimony constant.

#### 1.1.2. Expert Witness Gender

The impact of these expertise cues might depend, however, on the expert’s gender. Gender is an important cue because it does not actually relate to task performance but is nonetheless often used to infer how a person will perform (Bunderson, 2003). Research has demonstrated that women are less influential than men in groups, even when the task was stereotypically female (Propp, 1995), when everyone had equal access to the relevant information (Strodtbeck & Mann, 1956), or when a woman was, in fact, the most expert member of the group (Thomas-Hunt & Phillips, 2004). Even more concerning, this body of work suggests that not only is women’s expertise not recognized or valued as much as men’s, but it may also have a damaging effect. Women with greater expertise (defined as on-the-job experience and education) have been rated by their peers as less expert (Thomas-Hunt & Phillips, 2004), and their opinions were less relied upon in problem-solving tasks (Sonnentag & Volmer, 2009) relative to women with less expertise.

These studies suggest that gender may interact with other markers of expertise, such as education and on-the-job experience. However, these previous studies were not experimental, and it was not always clear what specific credentials the women had—gaps that our studies address. Further, the research reviewed above was conducted on teams of workers, which presents a different dynamic than listening to a testifying witness, who has been recognized as an expert by the court.

Research on the impact of an expert witness’s gender is mixed. In some studies, male expert witnesses are evaluated more favorably than female expert witnesses (Larson & Brodsky, 2014)—especially when both are portrayed as having low likeability or knowledge (Neal et al., 2012). However, some studies have found that participants view female experts as more likeable—though less credible—than male experts (Kipoulas et al., 2024). Evidence complexity has also played a contradictory role in predicting the influence of male (versus female) experts: in some cases female experts’ testimonies have less influence on case judgments than male experts’ when the evidence is complex (Schuller et al., 2005), though in others, the opposite pattern is true—female experts are more influential than male experts when evidence complexity is high, compared to when it is low (Maeder et al., 2012). This study also found that male jurors viewed female experts more favorably (and no effect for female jurors), which could hint at potential social desirability concerns surrounding exhibiting bias towards female experts in experimental settings.

Repeatedly, however, research demonstrates a favorability for experts who testify in domains that are stereotypically “consistent” with their gender (Schuller et al., 2005). Female experts are more influential when testifying in stereotypically “female” domains (e.g., battered women defense, Terrance et al., 2013; child custody, Schuller et al., 2005) and less influential when testifying in stereotypically “male” domains (e.g., the automotive industry; McKimmie et al., 2004). Further, aligned with stereotypes about women as lower in competency (Fiske et al., 2002), people responded more favorably towards female experts who used less complex language than females or males who used more complex language (McKimmie et al., 2013)—something that may be difficult to achieve when the expert is tasked with testifying about novel, complex scientific methods that require complex explanations. There are also a few studies that demonstrate that the gender of an expert witness had no effect on case judgments (Neal et al., 2012; Parrott et al., 2015) or in non-legal contexts (e.g., Holtgraves & Lasky, 1999). Studies 2 and 3 will test the interactive effect of expertise cues (i.e., education, direct contact with evidence) and the expert witness’s gender on mock jurors’ perceptions of their expertise and verdicts to further clarify the impact of expert gender in the courtroom.

### 1.2. The Impact of Explanation Satisfaction on Evaluations of Experts

In contrast to peripheral source characteristics (e.g., gender), a factor that should influence the evaluation of an expert is the explanation itself—particularly how satisfying judges and juries find the expert’s explanation. For instance, an expert who gives a more satisfying explanation of a scientific technique or piece of evidence might fairly be considered a “better” expert than one whose explanation is lacking. However, to our knowledge, no research has explored whether a juror’s subjective assessment of how satisfying an explanation is can influence evaluations of a witness’s expertise and the evidence itself.

Defining explanation satisfaction is a somewhat tricky task because satisfaction is an inherently subjective measure—what makes an explanation *satisfying* to one juror may not be the same thing that makes an explanation satisfying to another juror. It might be helpful to think of explanation satisfaction in a similar way to credibility. Expert credibility is a subjective construct: it is how much a juror, for example, perceives the trustworthiness and expertise of an expert witness. In some studies, researchers measure how credible jurors find an expert and demonstrate its importance in predicting the jurors’ decisions in line with that expert (e.g., Cramer et al., 2009). Researchers have also manipulated many factors that they think might, theoretically, influence credibility ratings—ranging from varying biographical information (e.g., gender, Larson & Brodsky, 2014; credentials, Pornpitakpan & Francis, 2000) or trustworthiness cues (e.g., potential bias; Susmann & Wegener, 2023). Though credibility is more relevant to the expert, we conceptualize explanation satisfaction as a similar construct, but about their testimony specifically. Both constructs could be influenced by different factors for different jurors. That is, like explanation satisfaction, credibility is a factor that jurors can agree on (i.e., this expert seems credible, their explanation was satisfying), without precisely being able to define why exactly they came to this conclusion or what they relied on to make that determination.

Thus, in all of our studies, we began our novel investigation of the role of explanation satisfaction in juror decisions by asking mock jurors to evaluate how satisfying they personally found the explanation provided by the expert. This question has been used in past literature to assess the perceived quality of an explanation (Bonawitz & Lombrozo, 2012; Frazier et al., 2009; Giffin et al., 2017; Liquin & Lombrozo, 2018). The advantage of this approach is that participants know how satisfied they are, even when they might not be able to assess whether an explanation was “correct” or not (because, by definition, they are hearing something that they do not have enough expertise to accurately evaluate; Wilkenfeld et al., 2016). Satisfaction can be understood as a person’s subjective belief that they received a “good” explanation or received the information they need to make a decision, regardless of whether or not this would be judged objectively true by people knowledgeable in the field.

Our first goal was to quantify the role that explanation satisfaction plays in whether mock jurors selected the verdict in line with the expert’s testimony. We also tested the role of explanation satisfaction in the context of other source cues about the expert themselves. That is, the average juror may not trust their ability to understand and fairly evaluate novel or complex scientific testimony and thus rely on source characteristics, such as the expert’s education, to help them decide whether to integrate the expert’s findings into their verdict decision. In support of this possibility, some research suggests that jurors rely more on source characteristics (e.g., trusting an expert with more, versus less, impressive credentials) as the complexity of the scientific techniques increases—suggesting a deferral to these sorts of expert cues (Cooper et al., 1996; Koehler et al., 2016). We wanted to explore whether explanation satisfaction was influenced by who the expert was (i.e., their gender, credentials, or contact with evidence) or if it was a relatively stable subjective impression regardless of who was delivering it.

Finally, in Study 3, we manipulated factors that we predicted would impact satisfaction. Because explanation satisfaction is so subjective, many different things might shape jurors’ subjective experiences of explanation satisfaction. Though research has found that simple explanations are generally seen as good explanations (Lombrozo, 2016), simple explanations of scientific evidence are not always possible in court. In fact, the few studies manipulating the complexity of an expert’s language have found no clear and consistent evidence that simpler testimony is favored or more impactful (Cooper et al., 1996; Schuller et al., 2005)—although, as noted, this might vary depending on the expert’s gender (McKimmie et al., 2013). These findings might also reflect the fact that trial testimony can be complex, and participants might feel a simple explanation is incomplete when discussing scientific evidence.

Alternatively, jurors may use features of the explanation itself to decide if it is satisfying, and this may override reliance on source characteristics. For instance, jurors’ reliance on peripheral source characteristics (like gender, education, etc.) might increase with increases in complexity, but this process could be mitigated when jurors hear a more satisfying, clear explanation. That is, it is possible that heuristic reliance on source factors could be reduced, even for complex and novel evidence, if an expert provides a clear, satisfying explanation. We test this possibility in Study 3 by experimentally manipulating the expert’s testimony to tap into possible features that could impact explanation satisfaction—the confidence, clarity, and fluency of the expert testimony, which have been related to satisfaction in other fields (e.g., Packard & Berger, 2021).

Further, explanation quality could interact with other expert source cues to differentially impact perceptions of their expertise. For example, experts with more advanced degrees or prestigious credentials (i.e., higher status experts) may only be favored so long as they provide a high-quality explanation. That is, we may see a potential penalty effect where explanation quality might interact with source characteristics, such that a high-status speaker might be penalized more for providing a bad explanation than a low-status speaker (Bohner et al., 2002). According to the shifting standards theory (Biernat & Manis, 1994), mock jurors may hold higher expectations for experts who are of higher status and evaluate their testimony relative to these expectations, compared to lower status experts who are evaluated against lower expectations. This theory would posit that group members are evaluated relative to the status of their own group—not across groups. Thus, experts who have achieved impressive credentials may be expected to provide a higher quality, “better” explanation or risk not meeting mock jurors’ expectations for that expert. In contrast, lower status experts may have a lower threshold to meet for what constitutes a good explanation *for their group* and would not be penalized so long as their explanation is sufficient for meeting this expectation. In these studies, we considered whether high-status speakers (i.e., male experts, experts with PhDs) would be penalized more for less satisfying explanations relative to lower-status speakers (i.e., female experts, experts with BAs).

### 1.3. The Current Research and Hypotheses

In three mock jury experiments, we assessed the degree to which mock jurors evaluated an expert witness’s explanation of touch DNA evidence and how this differed under three different source characteristics: education (Studies 1–3), level of direct contact with evidence (Studies 1–2), and expert gender (Studies 2–3). In Study 3, we also experimentally manipulated aspects of the expert’s communication style that we predicted would influence explanation satisfaction. In all three studies, we assessed mock jurors’ perceptions of the expert witness’s expertise, verdict, and satisfaction with the expert’s explanation.

**Hypothesis** **1.** *Mock jurors will rate an expert witness with more education (i.e., Doctor of Philosophy; PhD) as having greater expertise and more satisfying explanations than an expert witness with less education (i.e., Bachelor of Arts; BA). Greater expertise will predict more verdicts consistent with the expert witness’s testimony (i.e., more guilty verdicts).*

**Hypothesis** **2.** *Mock jurors will rate an expert witness with more direct contact with the evidence (i.e., lab technician) as having more expertise and more satisfying explanations than an expert witness with no direct contact with the evidence (i.e., lab supervisor). Greater expertise will predict more verdicts consistent with the expert witness’s testimony (i.e., more guilty verdicts).*

**Hypothesis** **3.** *Mock jurors who read an explanation that is more satisfying (relative to less satisfying) will have higher ratings of the witness’s expertise. This will, in turn, predict an increase in verdicts in line with the expert’s testimony (i.e., guilty verdicts).*

**Hypothesis** **4.** *Mock jurors will rate female experts as having less expertise and less satisfying explanations than male experts. Lower expertise ratings for female expert witnesses will, in turn, predict less of an impact on verdicts than male experts.*

**Hypothesis** **5.** *Mock jurors will penalize experts with higher status (i.e., experts with a PhD, male experts) for less (versus more) satisfying explanations by having decreased perceptions of their expertise, decreased satisfaction with their explanation, and fewer guilty verdicts; in contrast, perceptions of lower status experts will be less affected by the quality of their explanation.*

## 2. Study 1

In Study 1, we randomly assigned mock jurors to read testimony from a male expert who either (a) had a PhD or a BA in molecular biology, and (b) was the technician who had collected and processed the touch DNA evidence (i.e., more direct contact with the evidence) or the lab supervisor who had only reviewed a technician’s report (i.e., no direct contact with the evidence). We also included an additional exploratory condition with an expert who was hired by one side to review the technician’s report and offer their opinion. Mock jurors may view a hired expert, who is even more removed from contact with the evidence than the supervisor, less favorably because of the common sentiment of this type of expert as a “hired gun” (Cooper & Neuhaus, 2000) who may be more likely to deliver a one-sided testimony. This design allowed us to independently consider the contribution of contact with the evidence and education to assess whether either—or both—exerted influence on mock jurors’ evaluations of the experts and their verdicts.

### 2.1. Study 1 Method

#### 2.1.1. Participants and Design

Our final sample consisted of 464 adults who were recruited through Amazon Mechanical Turk and compensated for their participation. Participants ranged from 19 to 71 years old (*M* = 35.68, *SD* = 9.85) and were predominantly male (59.7%, 39.7% female, 0.6% other/prefer not to specify). Sample size was determined based on an a priori power analysis of previous expert witness studies (i.e., Giffin et al., 2017) to be approximately 100 participants per cell. In all studies, only individuals who (a) had an IP address within the United States, (b) had a prior approval rating of 95% or higher on previous tasks, and (c) had completed at least 50 tasks were eligible to participate in this study. The approval rating and number of tasks have been found to increase the quality of online data collected (Burhmester et al., 2011; Burhmester et al., 2018). Participants were also required to indicate that they were United States citizens and 18 years or older before participating in the study in order to ensure that participants met the basic requirements of jury service. An additional 38 participants were recruited but were excluded for failing at least one of three possible attention and manipulation questions designed to check whether they had been reading the trial stimuli and questions carefully. We include additional information about power analyses, checks, and statistical assumptions in the Supplementary Materials (pp. 8–9).

Our design was a 2 (expert education: *BA, PhD*) × 2 (expert experience: *technician, supervisor*) + 1 (PhD and hired to testify) between-subjects design.

#### 2.1.2. Materials and Procedures

Trial stimulus materials were based on a real case of a female college student who was murdered in the 1980s. Her car was found empty next to the side of the highway, and her body had been thrown off a nearby highway overpass. A highway patrol officer was eventually charged with the murder. The facts and testimony were collected from published accounts of the case and trial (Cantlupe & Petrillo, 1991; Figueroa, 2018). The names were changed, and some material was updated to more closely match current forensic practices and standards.

All mock jurors first read background information and short summaries of testimony from eleven witnesses, including the detectives investigating the case and the patrol officer accused of the crime. All testimony summaries included information elicited by both the prosecution and the defense to simulate direct and cross-examination in an actual trial. This trial material was identical in all conditions. See Supplementary Materials (pp. 3–6) for full stimuli.

Mock jurors were then randomly assigned to one of the five expert witness conditions. In all the conditions, the expert was a man, and mock jurors were not told which side had hired the expert, though based on the content of the testimony (i.e., the DNA matched the defendant), they could have inferred the expert was hired by the prosecution. The expert was first questioned by the prosecution and then the defense. Mock jurors in each condition were presented with a photograph and short description of their expert containing information on his education and job position. All descriptions were accompanied by the same stock photograph of a White, middle-aged man. The photograph was included to control assumptions about race or age without having to specifically draw attention to these factors.

After being introduced to the expert, mock jurors read a transcript of the expert’s testimony. First, the prosecution established the witnesses’ credentials by asking them about their education and experience with the evidence based on the specific experimental condition. Then the prosecution had the expert testify about the evidence, how the evidence was collected, and the procedure used to analyze it. In all conditions, the expert testified about touch DNA found on the handle of the victim’s car door. After the initial information specifically provided about the expert’s education and experience, the testimony of the witnesses was identical except when the supervisor and hired expert made it clear that they had not had any personal contact with the evidence and that the technician collected the evidence. In each condition, the expert concluded that the touch DNA evidence left at the scene belonged to the defendant. Thus, a guilty verdict would be consistent with the expert’s testimony. Next, the defense cross-examined the witness, and, finally, the prosecution asked re-direct questions. A full transcript of the testimony can be found in Supplementary Materials (pp. 3–6).

**Education manipulation.** For the education manipulation, mock jurors were randomly assigned to read that the expert either has a PhD in molecular biology or had a BA in molecular biology. Mock jurors were also told that the expert with a BA had completed four years of schooling, while the expert with a PhD had completed ten. This manipulation was reinforced by referring to the expert as either Dr. Jones or Mr. Jones throughout the description and testimony in the transcript.

**Experience manipulation.** For the evidence experience manipulation, mock jurors were randomly assigned to read either (a) “[Dr./Mr.] Jones is the laboratory technician who collected and processed the touch DNA evidence from the victim’s car” (i.e., the direct contact with the evidence condition), (b) “[Dr./Mr. Jones] supervises the lab that handled the touch DNA evidence from the victim’s car.” (i.e., the no-evidence-contact condition), or (c) “Dr. Jones is a researcher specializing in DNA. He has reviewed the lab report about the touch DNA evidence found on the victim’s car” (i.e., the no evidence contact/hired expert condition). It was again made clear to the mock jurors in the expert testimony that neither the supervisor nor the hired expert had ever handled the evidence directly.

#### 2.1.3. Measures

After reading their assigned transcript, mock jurors completed measures in the order presented below, though the order of the expertise and explanation satisfaction measures was randomized.

**Verdict.** Mock jurors were first asked to give their verdict on whether the defendant, Officer Doyle, was guilty of the murder of the young girl. The language in the verdict question was based on the first-degree murder statute (California Penal Code, 2025) and the reasonable doubt charge for the state of California (CALCRIM 103)—where the crime took place. Mock jurors could then choose either *not guilty* or *guilty* on the first-degree murder verdict option. They were also asked to indicate how likely they believed it was that Officer Doyle committed the murder on a scale of 1–100% in 1% increments; however, given that the results were largely the same as the dichotomous verdict, we do not report those analyses further.

**Expertise ratings.** Mock jurors were asked questions, in a randomized order, about their impressions of the witness who testified about touch DNA. Mock jurors responded to the items on 7-point Likert scales summarized in Table 1^1^.

We conducted an exploratory factor analysis using a principal axis factoring extraction with a Promax rotation, which revealed a single-factor structure (i.e., only one eigenvalue above 1, 3.51, and all factor loadings above 0.76). A reliability analysis confirmed that the scale had good reliability (Cronbach’s α = 0.89), so we calculated a mean *general expertise scale* score for each mock juror and found that across conditions, mock jurors believed their expert was high in expertise (*M* = 6.04, *SD* = 0.95).

**Explanation Satisfaction.** Mock jurors were also asked about the quality of the explanation of touch DNA offered by the expert witness. Mock jurors indicated “How satisfying was [Dr./Mr.] Jones’ explanation of touch DNA?” on a 1 (*Not at all satisfying*) to 7 (*Very satisfying*) Likert scale. Overall, mock jurors had high satisfaction with their expert’s evidence explanation (*M* = 5.99, *SD* = 1.11).

### 2.2. Study 1 Results

Verdicts were analyzed with logistic regression. All other continuous variables were analyzed with a 2 (expert education: *PhD*, *BA*) by 2 (expert experience: *technician, supervisor*) plus one (*hired expert*) between-subjects ANOVA, unless specified otherwise. The full design effects, including the *hired expert* condition, are reported fully in the Supplementary Materials (pp. 9–11).

#### 2.2.1. Explanation Satisfaction

An ANOVA found no significant effects of the manipulations on explanation satisfaction, all *F*s ≤ 2.13, all *p*s ≥ .15. In other words, no source characteristics of the expert (i.e., education, experience) had an impact on mock jurors’ ratings of how satisfying their explanation of the evidence was; mock jurors were equally satisfied with the expert’s explanation regardless of who was delivering it.

#### 2.2.2. Expertise Ratings

Hypotheses 1 and 2 predicted that an expert’s experience and education would increase ratings of their expertise. We performed a between-subjects ANOVA on expertise scale scores. Consistent with Hypothesis 1, education had a significant main effect on perceptions of the witness’s expertise, *F*(1, 459) = 6.95, *p* = .01, *η_p_*^2^ = .02, such that mock jurors believed experts with a PhD had higher expertise than experts with a BA (Table 2).

However, contrary to Hypothesis 2, we found no effect of experience, *F*(2, 459) = 1.36, *p* = .26, *η_p_*^2^ = .01, or an interaction between experience and education, *F*(1, 459) = 0.08, *p* = .79, *η_p_*^2^ = .00. That is, mock jurors did not see an expert who had direct contact with the evidence as being more of an expert than one who was more removed from the evidentiary collection process. These characteristics did not interact to differentially predict expertise scores.

Hypothesis 3 examined whether explanation satisfaction would predict more favorable expertise ratings, and our results support this prediction. A linear regression found that mock jurors’ being more satisfied with the witness’s explanation was associated with rating the witness as having more expertise, *B* = |0.60|, *SE* = 0.02, *p* < .001, *R*^2^ = .49. This suggests that when mock jurors perceive an expert as having more expertise, they also believe that the expert’s explanation is more satisfying. We will test the possibility of a causal explanation in Study 3.

#### 2.2.3. Verdicts

Hypotheses 1, 2, and 3 predicted that an expert’s education, experience, and mock jurors’ satisfaction with the explanation they offered would have an impact on the verdicts that mock jurors rendered. To test these predictions, we first ran a logistic regression with experience (1 = hired expert, 2 = supervisor, 3 = technician), education (0 = BA, 1 = PhD), and mock jurors’ explanation satisfaction, as well as all interaction terms predicting dichotomous verdicts. We found that none of the two-way or three-way interactions were significant: all *B*s ≤ |−1.93| and all *p*s ≥ .26. In examining the initial main-effects-only step, mock jurors’ satisfaction with the witness’s explanation was associated with being more likely to choose a guilty verdict in line with the expert’s testimony: *B* = |0.63|, *SE* = 0.10, Wald = 37.00, *p* < .001, and *OR* = 1.88. With each increase of one satisfaction scale point, mock jurors were almost twice as likely to vote guilty (i.e., in line with the expert’s testimony). The expert’s experience was also a significant predictor—*B* = |0.29|, *SE* = 0.14, Wald = 4.32, *p* = .04, and *OR* = 1.34—such that as the experts increased in proximity to the evidence, mock jurors were 1.34 times more likely to be influenced by the expert’s testimony (i.e., vote guilty). Education did not have a significant effect: *B* = |0.35|, *SE* = 0.21, Wald = 2.69, *p* = .10, and *OR* = 1.41.

### 2.3. Study 1 Discussion

Despite mock jurors finding experts with higher educational credentials (i.e., a PhD versus a BA in molecular biology) to have more general expertise, credentials did not impact verdicts. Instead, mock jurors were more influenced (i.e., voted guilty more often) when they heard from an expert with more direct contact with the evidence than by experts who were more distant—even though they did not explicitly rate the *Technician* to have more expertise than the *Supervisor*. This provides partial support for both Hypotheses 1 and 2.

That is, for Hypothesis 2, we have an outcome effect without a clear mechanism: more direct contact with the evidence led the expert to have more influence on verdicts without necessarily being seen as having more expertise. One possible explanation is that these expertise questions were not specific to the case about which the expert was testifying. In the *Technician* conditions, the expert had contact with the evidence in this *specific* case, but the expertise questions referred to the expert’s *general* knowledge, helpfulness, etc. If we had asked about the witness’s *case-specific* expertise, we might have seen perceived expertise explain the effect of evidence contact on verdicts—a possibility we test in Study 2.

The results of Study 1 offered support for Hypothesis 3. We found that being more satisfied with the witness’s explanation predicted both rating the witness as having greater expertise and a greater likelihood of voting in line with the expert’s opinion (i.e., voting guilty). Interestingly, neither the expert’s education nor experience predicted how satisfied mock jurors were with his explanation—suggesting that satisfaction with the explanation is a construct independent from expertise source cues. Perhaps mock jurors were able to evaluate the explanation without being influenced by credentials or experience.

## 3. Study 2

Study 1 demonstrated that mock jurors perceive expert education and contact with the evidence differently. An expert’s education predicted perceived expertise (but not verdicts), whereas an expert’s level of direct contact with the evidence predicted verdicts in line with the expert’s testimony (but not expertise). Perhaps this is because mock jurors recognize that an expert with more education may have more general knowledge, though not necessarily in this specific case. The opposite might be true for an expert who had more direct contact with the evidence: their level of contact with the evidence may be directly applicable in this case (e.g., should be influential), but does not serve as a cue to their overall expertise in the field. Explanation satisfaction, however, predicted both expertise and verdicts.

Study 2 was designed to replicate these key findings of Study 1 and further investigate how and why evidence contact influences verdicts. It may be the case that the lack of difference in perceptions of expertise between the *technician* and the *supervisor* conditions is due to a measure that focuses more on expertise in general, rather than expertise in this specific case. Thus, we have added questions to independently identify relative perceptions of the expert’s specific versus general expertise. Additionally, because we did not find any differences between the hired expert and PhD condition (see Supplementary Materials, pp. 9–10), and for simplicity of design, we removed the hired expert condition from subsequent studies.

A secondary goal of Study 2 was to extend our research questions to explore the impact of an expert’s gender. Prior work on evaluations of expertise and gender suggests that people may not accurately evaluate women’s expertise, and, subsequently, women with more expertise may actually have less influence over decisions than women who have less expertise (Thomas-Hunt & Phillips, 2004; Sonnentag & Volmer, 2009). Further, compared to male expert witnesses, female expert witnesses tend to be rated less favorably and have less influence on juries (Larson & Brodsky, 2014; Maeder et al., 2012; McKimmie et al., 2004, 2013; Neal et al., 2012; Schuller et al., 2005). Hypothesis 4 predicts that female experts will be evaluated more negatively and, in turn, have less influence on verdicts than male experts who present the same exact testimony—particularly given we are testing this effect in a stereotypically male domain (i.e., DNA evidence is within the STEM domain, in which women are underrepresented; National Center for Science and Engineering Statistics, 2024).

### 3.1. Study 2 Method

#### 3.1.1. Participants and Design

Our final sample included 651 adults who were recruited through Amazon Mechanical Turk and compensated for their participation. Participants ranged from 18 to 71 years old^2^ (*M* = 35.64, *SD* = 10.88) and were relatively evenly split between male and female (52.6% male, 46.9% female, 0.5% other/prefer not to specify). The sample size increase was made to accommodate the additional four cells of the gender manipulation. Participation in the experiment was restricted using the same criteria as in Study 1, and anyone who participated in Study 1 was not eligible to participate in Study 2. We excluded an additional 152 participants (18.9%) who were tested but were removed for failing attention and manipulation checks.

Mock jurors were randomly assigned to one of eight conditions based on a 2 (expert gender: *male*, *female*) by 2 (expert experience: *technician*, *supervisor*) by 2 (expert education: *BA*, *PhD*) between-subjects design.

#### 3.1.2. Materials and Procedures

The background information and testimony given were identical to Study 1, except that masculine and feminine pronouns were used where appropriate (e.g., “Ms. Jones”). The expert descriptions were also identical to those used in Study 1, except that half the conditions were accompanied by a stock photograph of a middle-aged, white woman and used feminine pronouns, while the other half were accompanied by the same stock photograph of a middle-aged, white man and used masculine pronouns, identical to Study 1.

#### 3.1.3. Measures

After reading the background and testimony of the randomly assigned expert, mock jurors answered the same questions, in the same order, as in Study 1—with one addition. Because the original expertise items more closely measured “general” expertise, we added a set of three items that more directly gauged “specific” expertise. The three questions measured mock jurors’ perceptions of case-specific expertise on 7-point Likert scales and are reported in Table 1 and Table 3. This allowed us to measure mock jurors’ perceptions of their expert’s “general” (Cronbach’s α = 0.85; *M* = 6.15, *SD* = 0.84) and “specific” (Cronbach’s α = 0.81; *M* = 5.76, *SD* = 1.03) expertise.

### 3.2. Study 2 Results

Verdicts were analyzed with logistic regression. All other continuous variables were analyzed with a 2 (expert gender: *male*, *female*) by 2 (expert education: *BA*, *PhD*) by 2 (expert experience: *technician, supervisor*) between-subjects ANOVAs, unless specified otherwise. While we only report significant effects here, full reports of all tests are available in the Supplementary Materials (pp. 12–14).

#### 3.2.1. Explanation Satisfaction

**Figure 1:**
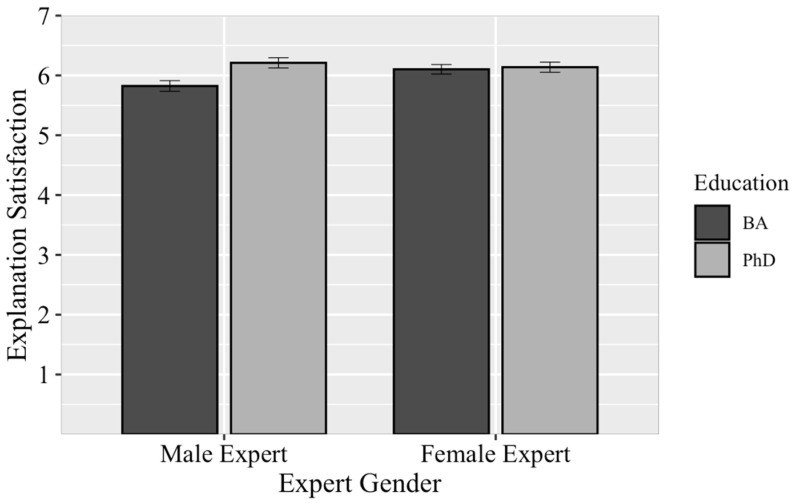
Explanation satisfaction as a function of expert gender and expert education. Note. Explanation satisfaction ranges from 1 (*not at all satisfying*) to 7 (*very satisfying*). Error bars are +/− 1 SE.

Unlike in Study 1 and in support of Hypothesis 2, we found that witness’s education level had a significant main effect on explanation satisfaction—*F*(1, 643) = 6.10, *p* = .01, and *η_p_*^2^ = .01—with mock jurors rating the same explanation as being more satisfying when offered by an expert with a PhD than when offered by an expert with a BA. This main effect was qualified, however, by a significant interaction between expert gender and education: *F*(1, 643) = 4.37, *p* = .04, and *η_p_*^2^ = .01 (Figure 1). Despite having given the exact same testimony, the *male BA* expert was rated as giving a less satisfying explanation than the *male PhD*: *F*(1, 643) = 10.11, *p* = .002, *η_p_*^2^ = .02. In contrast, mock jurors rated the *female BA* expert and *female PhD* expert as having offered a similarly satisfactory explanation: *F*(1, 643) = 0.08, *p* = .79, and *η_p_*^2^ = .00; this maps onto the penalty theory in Hypothesis 5 (i.e., higher status experts, like men, might be penalized for having lesser education credentials). There were no other significant main effects or interactions: all *F*s ≤ 1.90 and all *p*s ≥ .17.

#### 3.2.2. General Expertise Ratings

Replicating Study 1 and supporting Hypothesis 2, the witness’s education had a significant main effect on generalized expertise—*F*(1, 643) = 12.93, *p* < .001, and *η_p_*^2^ = .02—such that *PhD* experts were rated as having significantly more general expertise than *BA* experts. No other main effects or interactions were significant: all *F*s ≤ 3.65 and all *p*s ≥ .06.

#### 3.2.3. Case-Specific Expertise Ratings

In contrast to general expertise ratings (and as expected), education did not have a significant effect on *case-specific* expertise ratings: *F*(1, 643) = 0.55, *p* = .46, and *η_p_*^2^ = .001. Instead, there was a main effect of experience; a lab technician who had more contact with the evidence (*M* = 5.90, *SD* = 0.96) was rated as having significantly more case-specific expertise than the lab supervisor (*M* = 5.62, *SD* = 1.08): *F*(1, 643) = 12.32, *p* < .001, and *η_p_*^2^ = .02. We also found a main effect of gender such that mock jurors perceived female witnesses (*M* = 5.84, *SD* = 0.98) as having more case-specific expertise than male witnesses (*M* = 5.67, *SD* = 1.08): *F*(1, 643) = 4.33, *p* = .04, and *η_p_*^2^ = .01. No interactions were significant: *F*s ≤ 0.88 and all *p*s ≥ .35.

In support of Hypothesis 3, linear regressions revealed that mock jurors’ explanation satisfaction was again associated with rating the expert as having more general expertise—*B* = |0.55|, *SE* = 0.02, *p* < .001, and *R*^2^ = .50—and also as having more specific expertise—*B* = |0.49|, *SE* = 0.03, *p* < .001, and *R*^2^ = .27. The more satisfied the mock juror was with the expert’s explanation, the higher they rated the expert on both general and specific expertise.

#### 3.2.4. Verdicts

We conducted a logistic regression with expert gender (0 = *male*, 1 = *female*), experience (0 = *technician*, 1 = *supervisor*), education (0 = *BA*, 1 = *PhD*), mock jurors’ satisfaction with the expert’s explanation, and all interactions predicting verdicts. None of the interactions were significant: *B*s ≤ |4.40| and all *p*s ≥ .11. The main-effects-only step partially replicated Study 1 in that it revealed that explanation satisfaction was again a significant predictor of verdicts—*B* = |0.43|, *SE* = 0.09, *p* < .001, and *OR* = 1.53—with more satisfying explanations predicting more guilty verdicts in line with the expert’s opinion. Expert gender (*B* = |0.10|, *SE* = 0.16, *p* = .56, *OR* = 1.10), education (*B* = |−0.14|, *SE* = 0.16, *p* = .41, *OR* = 0.87), and experience (*B* = |0.04|, *SE* = 0.16, *p* = .81, *OR* = 1.04) were all non-significant predictors of verdicts.

### 3.3. Study 2 Discussion

Study 2 again provided mixed support for Hypotheses 1 and 2. Replicating Study 1, the expert’s education increased perceptions of general expertise, while we discovered that more direct experience with the evidence increased perceptions of case-specific expertise. Interestingly, this suggests that jurors may be quite discerning when evaluating experts. Potential jurors were able to recognize that education and evidence contact yield different types of knowledge and thus could differentiate between different expertise cues. Yet, these types of expertise did not translate into having more influence on verdicts.

Although having direct experience with the evidence did not have a significant impact on verdicts in Study 2, this experiment does shed some light on how mock jurors view the two experience conditions. The *technician* was rated significantly higher on case-specific expertise than the *supervisor*, but the two conditions did not vary in ratings of general expertise. This suggests, as predicted, that mock jurors recognized that the *technician* had contact with the evidence in this case, which may lend him or her more expertise than the more removed *supervisor* when testifying about this case specifically, but not in general.

Study 2 replicated Study 1’s support for Hypothesis 3: the degree to which mock jurors were satisfied with the expert’s explanation significantly predicted both perceiving the witness as having more expertise and a greater likelihood of being influenced by the expert’s opinion in their verdict choice. In fact, when expert gender, type of experience, education, and mock jurors’ satisfaction with the expert’s explanation were all entered into a binary logistic regression predicting verdicts, explanation satisfaction was the only significant predictor.

Hypothesis 4, regarding a potential gender bias against female experts, was directly contradicted. Expert gender did not have an effect on verdicts, and to the extent that expert gender had an effect on perceptions of expertise, female experts were rated as having more case-specific expertise than male experts. Perhaps this occurred because mock jurors who saw a female expert engaged in shifting standards (e.g., rating a female expert as having higher levels of expertise *for a woman*; Biernat & Manis, 1994) or re-fencing (e.g., this female expert is highly competent and an exception to other stereotypes about women; Allport, 1954). Mock jurors were also similarly satisfied with female experts’ explanations and rated them as having similarly high levels of general expertise regardless of their education level; in contrast, male experts were penalized on these factors when they had a BA relative to a PhD. It could be that mock jurors recognize it has historically been more difficult for women to obtain advanced degrees and credentials in STEM fields, and so perhaps mock jurors who see a woman in a position of expertise (even if with a less advanced degree) also view them as particularly intelligent or persistent, relative to men. This may explain why both female BAs and female PhDs were rated as highly as the male PhD, and all higher than the male BA, despite reading the same expert witness testimony.

One limitation of the previous two studies was that explanation satisfaction was measured, rather than manipulated. Thus, we cannot conclude that explanation satisfaction has a causal effect on perceptions of expertise or verdicts. Further, mock jurors rated the expert’s explanation quite highly in both Study 1 (*M* = 5.99, *SD* = 1.11) and Study 2 (*M* = 6.07, *SD* = 1.09) on 7-point scales. In Study 3, we tested whether we could manipulate how satisfied mock jurors were when an expert reached the same conclusion and had the same basis for that conclusion but offered explanations in a more clear and detailed manner, relative to a less clear and detailed manner. This also enabled us to test whether higher status experts (i.e., male versus female, PhD versus BA) might be penalized more for offering a less satisfying explanation than lower status experts.

## 4. Study 3

The most consistent finding across studies thus far is the importance of explanation satisfaction, consistent with Hypothesis 3. In Studies 1 and 2, mock jurors’ satisfaction with the expert’s explanation was a significant predictor of both their perceptions of the expert’s expertise and their verdicts. Mock jurors’ ratings of how satisfied they were with the explanation were only once affected by who the expert was (Study 2)—suggesting that mock jurors’ satisfaction with the expert’s explanation seems to have an impact on their decisions that is largely independent of our manipulated source factors.

Study 3 was designed to expand these findings by experimentally manipulating the explanation to see if we could decrease explanation satisfaction and, in turn, cause a decrease in perceptions of expertise and influence on verdicts. Further, prior research has found that poor explanations from high-prestige sources were rated more harshly than when those same poor explanations were given by a less prestigious source (Bohner et al., 2002). However, the high and low prestige sources in the previous study were vastly different from each other, a college professor and a high school student, so it remains to be seen if less extreme differences in prestige (that would both be qualified as experts in court) would produce these effects—especially when all have been deemed experts by the court. The explanation manipulation enabled us to test whether a relatively higher-status expert (i.e., men, PhDs) would be penalized more for a relatively worse explanation than would lower-status experts (women, BAs).

Previous work would suggest that female experts would be seen as relatively lower status than male experts, so any higher penalty should go to the male expert. This might also, in part, explain the effect we saw in Study 2, wherein the male expert was rated as having significantly less expertise when he had a BA relative to when he had a PhD—a penalty that did not manifest for women. Perhaps the higher status expert (i.e., the male expert) was penalized more for lower levels of achievement. That is, mock jurors expected more of him due to his gender and the higher status it confers. Thus, in Study 3, we again manipulated gender and education to further explore this effect, as well as adding the explanation manipulation to see if one gender would be penalized more than the other for unsatisfying explanations.

Finally, rather than relying on only one male photograph and one female photograph, we employed stimulus sampling to make sure that gender effects were not due to the specific photographs chosen for the experts in Study 2.

### 4.1. Study 3 Method

#### 4.1.1. Participants and Design

Our final sample in Study 3 was 738 adults who participated in the study through Amazon Mechanical Turk for monetary compensation. Participants ranged from 18 to 74 years old (*M* = 35.45, *SD* = 10.86) and were approximately split between male and female (54.5% male, 45.1% female, 0.1% other/prefer not to identify). An additional 88 participants were tested (10.7%) but were excluded for failing attention and/or manipulation checks. Participation in the experiment was restricted using the same criteria as in Studies 1 and 2. Participants who had participated in either of the prior experiments were not eligible for Study 3.

Mock jurors were randomly assigned to one of eight conditions based on a 2 (expert gender: *male*, *female*) by 2 (expert education: *BA*, *PhD*) by 2 (expert explanation: *less satisfying*, *more satisfying*) between-subjects design. The experience-type manipulation was dropped in Study 3 for power concerns and because it did not have a consistent impact on explanation satisfaction, expertise ratings, or verdicts across Studies 1 and 2. All experts in Study 3 were described as technicians.

#### 4.1.2. Materials

**Explanation Satisfaction Manipulation.** The background information, witness summaries, and expert testimony in the *more satisfying* explanation condition were identical to those used in Studies 1 and 2 because these materials had received such high satisfaction ratings from mock jurors. In the *less satisfying* explanation condition, the explanation contained the same essential information (i.e., the same conclusion and the same basis for the conclusion) as the *more satisfying* explanation, but we rewrote it with the goal of making the presentation style less satisfying. We wanted to ensure that while the explanation’s content was the same, the manner of presentation varied in several ways. Specifically, we made the expert’s presentation style more casual, less confident and straightforward, and relatively less clear because the language included more colloquial speech (e.g., saying “like” throughout) and a less fluid speech pattern. These factors were chosen because we believed that they would impact participants’ subjective ratings of experts’ explanation satisfaction. It was not our goal to pinpoint what makes a less satisfying explanation—this is the subject of an entire body of literature on its own (e.g., Sehl et al., 2024)—but to create a strong comparison comprising several factors that we believed *might* make it less satisfying without changing the content. We also kept the word count as similar as possible between the two conditions. The *less satisfying* explanation had 847 words while the *more satisfying* explanation had 856 words. The *less satisfying* explanation can be found in the Supplementary Materials (pp. 6–8).

After reading the background and the testimony of their assigned expert, mock jurors answered the same general expertise and verdict questions, in the same order, as in Studies 1 and 2. The general expertise items (Table 1) again formed a reliable scale: Cronbach’s α = 0.88. The case-specific expertise questions from Study 2 were removed because we also removed the contact-with-evidence condition in this study and believed that it would not be theoretically relevant with the existing conditions.

**Expert Gender Manipulation.** An additional change was that instead of using one stock photograph for the male expert and one for the female expert, mock jurors in the *Male* condition were randomly assigned to see one of three male stock photographs, and mock jurors in the *Female* condition were randomly assigned to see one of three female stock photographs. Each was of a middle-aged, White person, seen from the shoulders up, dressed in professional attire, and the photographs were edited to be the same size. This stimulus sampling was performed to ensure that any gender effects could not be due to idiosyncratic attributes or impressions of the two original photos used in Study 2.

Finally, we also measured the expert’s perceived warmth, competence, and persistence to test whether these factors explained the gender-by-education interaction from Study 2. These measures were presented among the general expertise scale items. However, to preview the results, we did not find statistically significant support for the gender-by-education interaction effects from Study 2. Thus, these measures were not able to provide insight into that effect, so we do not discuss them further but do provide preliminary analyses in Supplementary Materials (pp. 17–18). Other than the new explanation manipulation, additional questions, and stimulus sampling for the expert gender manipulations, the methods and procedures were identical to Study 2.

### 4.2. Study 3 Results

Verdicts were analyzed with logistic regression. All other continuous variables were analyzed with a 2 (expert gender: *male*, *female*) by 2 (expert education: *BA*, *PhD*) by 2 (expert explanation: *less satisfying*, *more satisfying*) between-subjects ANOVA, unless otherwise specified.

#### 4.2.1. Explanation Satisfaction

The explanation manipulation was the only variable to have a significant effect on measured explanation satisfaction: *F*(1, 730) = 20.35, *p* < .001, and *η_p_*^2^ = .03. Evidencing the effectiveness of the manipulation, mock jurors in the *more satisfying* condition provided significantly higher explanation satisfaction scores than mock jurors in the *less satisfying* condition—and this did not depend on any of the other manipulations. We did, however, find a three-way interaction between gender, education, and the explanation satisfaction manipulation on measured explanation satisfaction that approached significance (*p* = 0.051), which provides some potential support for the gender-by-education interaction presented in Study 2. We detail it fully in the Supplementary Materials (pp. 16–17) but refrain from discussing it here for brevity and because it was not significant.

However, it is worth noting that even in the *less satisfying* condition, the mean was still relatively high (*M* = 5.67, *SD* = 1.28) compared to (*M* = 6.06, *SD* = 1.08) in the *more satisfying* condition, and the effect size was not large. This is reassuring, in that it suggests mock jurors still felt that they received helpful information, even in the *less satisfying* explanation condition, but suggests the manipulation might be a relatively weaker comparison than originally intended—albeit a statistically significant one.

#### 4.2.2. General Expertise Ratings

We again found that mock jurors rated witnesses with a PhD as having more general expertise than witnesses with a BA—*F*(1, 730) = 5.91, *p* = .02, and *η_p_*^2^ = .01—replicating Hypothesis 1 from Studies 1 and 2. We also conceptually replicated our findings from Studies 1 and 2 and provided the first causal evidence that explanation satisfaction significantly increased perceived expertise: the *more satisfying* explanation significantly increased perceived expertise relative to the *less satisfying* explanation—*F*(1, 730) = 24.23, *p* < .001, and *η_p_*^2^ = .03. This suggests that delivering expert testimony that jurors find to be more satisfying can also impact their perceptions of the experts’ broader knowledge overall. There was no main effect of expert gender—*F*(1, 730) = 0.77, *p* = .38, and *η_p_*^2^ = .001—and no interactions were significant: *F*s ≤ 2.03 and all *p*s ≥ .15.

Using a linear regression, we replicated findings from Studies 1 and 2, that mock jurors’ subjective ratings of explanation satisfaction predicted expertise ratings: *B* = |0.58|, *SE* = 0.02, *p* < .001, and *R*^2^ = .50. The more satisfied the mock juror was with the explanation, the more favorably they rated the witness’s expertise.

#### 4.2.3. Verdicts

In both Studies 1 and 2, we found consistent support for Hypothesis 3, such that mock jurors’ satisfaction with the expert’s explanation was a significant predictor of verdicts. When expert gender, education, manipulated explanation satisfaction, and all interactions among the manipulations were entered into a binary logistic regression predicting verdicts, no interactions were significant: all *B*s ≤ |0.43| and all *p*s ≥ .13. The main-effects-only step of the model revealed that none of the manipulations had a significant effect: *B* ≤ |0.2| and *p* ≥ .17. A separate model with measured explanation satisfaction replicated the Studies 1 and 2 finding, however, that mock jurors’ ratings of their explanation satisfaction were again a significant predictor of verdicts: *B* = |0.36|, *SE* = 0.07, *p* < .001, and *OR* = 1.44.

#### 4.2.4. Mediation

We next tested the indirect effect of our explanation satisfaction manipulation on verdicts through perceived expertise using Hayes’ PROCESS Model 4 (Hayes, 2013). We ran a model with our manipulation of explanation quality—as more or less satisfying—predicting verdicts through the mock jurors’ ratings of general expertise. We found a significant indirect effect of manipulated explanation satisfaction on verdicts through mock jurors’ ratings of expertise: *M_indirect_* = 0.11, *SE* = 0.04, and 95% CI = [0.05, 0.20]. More specifically, mock jurors randomly assigned to read the more, versus less, satisfying explanation indeed reported that they perceived the expert witness to have greater expertise—*B* = |0.35|, *SE* = 0.07, *p* < .001, and 95% CI = [0.21, 0.49]—which in turn was associated with an increased likelihood of voting guilty: *B* = |0.32|, *SE* = 0.08, *p* < .001, and 95% CI [0.16, 0.48]. We conceptually replicate this finding with our measured explanation satisfaction variable as the focal predictor in the Supplementary Materials (p. 16).

### 4.3. Study 3 Discussion

Study 3 replicated several key results from Studies 1 and 2. In partial support of Hypothesis 1, the expert’s education again had a significant effect on perceived expertise: mock jurors again rated the PhD experts as having more general expertise than BA experts. However, (consistent with Studies 1 and 2), the expert’s education level did not impact verdicts. We again found support for Hypothesis 3, such that both mock jurors’ self-reported measure of explanation satisfaction as well as the experimental manipulation of explanation satisfaction increased the perceived expertise of the witness, and the former was associated with more guilty verdicts in line with the expert’s testimony. This finding is particularly interesting because it suggests that despite delivering the same information, mock jurors perceive experts who speak more clearly and confidently as having more general expertise than experts who do not—which can have implications for how much influence the expert has over verdicts. We found that the explanation satisfaction manipulation indirectly increased guilty verdicts through both mock jurors’ reports of perceived general expertise of the expert. That is, when mock jurors were randomly assigned to read a more (vs. less) satisfying explanation, they were more likely to be influenced by the expert’s opinion in their verdict decisions, through more favorable impressions of the expert’s general expertise. However, the effect of the explanation satisfaction manipulation was not strong enough to have a total effect on verdicts when perceptions of expertise were not accounted for in the overall model.

We did not find support for Hypotheses 4 or 5, in that expert gender did not affect any of our dependent variables. We did not replicate the Study 2 finding that *male BA* experts were penalized and rated as having less expertise than *male PhD* experts. However, in this study, we did include a few additional measures to try to explain the Study 2 gender-by-education effect that we do not report in the main text for brevity: perceptions of the experts’ warmth, competence, and perseverance (in Supplementary Materials, pp. 17–18). In summary, we found some evidence that male experts who had a BA were seen as less competent than male experts with a PhD, but mock jurors saw female experts as similarly competent—regardless of whether they had a BA or PhD. This suggests that perhaps it is not that women receive a boost for having achieved a certain level of education, but rather that men receive a penalty for not achieving it. While more data needs to be collected to confirm this interpretation, if supported, it bodes poorly for experts of all genders: education does not bolster women, but lack of it can damage perceptions of men’s intelligence.

## 5. Discussion

Scientific evidence is increasingly central to achieving justice in trials, and the ability of testifying experts to communicate science clearly and effectively is commensurately vital. It may also be important for jurors to be able to discriminate the content of the message from the messenger. The current studies investigated the independent impact of source characteristics that indicate expertise and jurors’ level of satisfaction with the expert’s explanation on evaluations of the expert and verdicts. We found that an expert’s education and level of hands-on experience with the evidence indeed had independent effects. Having a more advanced degree consistently increased mock jurors’ perceptions of the expert’s general expertise, while their proximity to the evidence directly increased perceptions of their case-specific expertise. Further, this demonstrates that jurors are able to differentiate between different kinds of expert characteristics and their resultant expertise. Perhaps jurors can recognize the value of experts who may have less impressive credentials but closer hands-on experience with the evidence. However, these source characteristics did not have a consistent effect on verdicts. Instead, the most consistent finding was the importance of jurors’ satisfaction with the expert’s explanation. Offering more satisfying explanations led to greater perceived expertise, which in turn predicted mock juror verdicts more in line with the expert’s testimony, relative to the verdicts of mock jurors who received less satisfying explanations—even when the same content and evidence were presented in both explanations.

### 5.1. The Key Role of Explanation Satisfaction

Hypothesis 3 predicted that explanation satisfaction was related to more verdicts in line with the expert’s testimony, and we found consistent support for this effect. Self-reported satisfaction with the expert’s explanation was the strongest predictor of verdicts in Study 1, and the only significant predictor in Studies 2 and 3. These assessments of explanation satisfaction were largely unaffected by the expert’s education, experience, and gender—suggesting that jurors’ satisfaction with the testimony was not due to the expert but rather the content of the testimony itself. In Study 3, we experimentally manipulated explanation satisfaction by varying features that we believed would impact jurors’ subjective satisfaction with the quality of the expert’s explanation of the same conclusion. We found that the explanation in the *more satisfying* condition increased perceptions of the expert’s general expertise, which significantly predicted verdicts in line with the expert’s testimony. Thus, considering self-report and the experimental manipulation together, the more satisfying an expert’s explanation, the more expertise mock jurors believed they had, and the more likely that mock jurors were to believe their testimony and find the defendant guilty. Importantly, to impact satisfaction, we did not materially change the content the expert conveyed—in all cases, they described their data collection, data processing, and conclusions. Rather, we chose to manipulate only *how* that explanation was presented by varying the degree to which the expert gave a clear explanation of the information relative to a more muddled one.

It should be noted that although our manipulation had an indirect effect on verdicts through perceived expertise, it did not have a total effect on verdicts—most likely due to the fact that our *more satisfying* explanation was significantly more satisfying, statistically speaking, but had a small effect size because both explanations were rated as relatively satisfying by mock jurors. A more extreme difference between an expert’s more versus less satisfying explanation would likely show a bigger relationship with verdicts. That such small changes in explanation presentation had any effect at all has important implications for the expertise and explanation literature, as well as practical legal significance. Prior studies have manipulated language complexity, finding that mock jurors preferred complex, jargon-heavy language from high-status speakers (Cooper et al., 1996; McKimmie et al., 2013; Schuller et al., 2005). Our studies show something different: holding content constant, experts can achieve greater persuasive power (by means of satisfying explanations) by simply focusing on presenting their evidence and conclusions in a more confident, clear, and detailed manner.

Our results are in line with the explanation literature. Studies on explanation preferences have previously found that people do prefer simple explanations over complex ones, even on more complex topics (Pacer & Lombrozo, 2017; Lombrozo, 2007; Lagnado, 1994). Our *more satisfying* explanation was designed to be as clear and as free from jargon as possible, considering the complex topic of testimony. Providing clarity and eliminating unnecessary jargon is one way to reach toward simplicity and, thus, greater satisfaction. Further, in our mock jurors, this greater satisfaction predicted greater endorsement of the testimony itself, as seen by the increased guilty verdicts in line with the expert’s testimony. The *more satisfying* manipulation also increased how much general expertise that mock jurors thought the expert had; speaking clearly and confidently—despite conveying the same information and conclusions—made mock jurors view the expert themselves as having greater expertise. Our studies sought to introduce concepts of complexity and clarity from the explanation literature to the study of expert witnesses, and our findings suggest that such a crossover is ripe for more investigation. Of interest to academics and practitioners, our findings suggest that experts need not rely on complexity and jargon to bolster their expertise and, in fact, may do better to strive for clarity. Because our test of the *less satisfying* explanation included more non-sequiturs and colloquial language, future research should test an explanation that is less satisfying, not because of its casual language but rather because of its over-the-top complexity. Investigating both ends of this spectrum will enlighten researchers on the specific relationship between complexity and explanation satisfaction.

We also explored the possibility that the characteristics of the expert could differentially influence how mock jurors evaluated that expert’s explanation. That is, an explanation from an expert with a PhD could be assumed to be better than one from an expert with only a BA, and this assumption might be reflected in mock jurors’ expertise ratings. This might be expected if, for instance, mock jurors were processing information about the expert heuristically (Petty & Cacioppo, 1986) or relied on outside cues about the expert because they did not feel competent to evaluate the substance of the explanation. However, we found limited evidence that this was the case. In Study 2, we did find that male experts who had a BA were rated as giving a less satisfying explanation than men who had a PhD (an effect not found for female experts), though, in general, education did not predict explanation satisfaction in Studies 1 or 3. Overall, it appears that our mock jurors judged the quality of the explanation independently of the source characteristics of the expert. It could be that mock jurors used a more analytical central processing approach to evaluate the evidence of a trial in determining innocence and guilt (Petty & Cacioppo, 1986). Though more research remains to be performed, our studies suggest that a better explanation has a persuasive power all its own.

While source characteristics had limited impact on explanation appraisals, explanation satisfaction did have a significant influence on how experts were judged. Hypothesis 3 predicted that people’s evaluations of an expert would be affected by their satisfaction with the expert’s explanation, and we found support for this hypothesis. Mock jurors randomly assigned to the *more satisfying* explanation rated the experts as having more general expertise than those who read the *less satisfying* explanation in Study 3—despite holding the evidentiary basis and conclusion constant. So, while we saw only a weak effect of source characteristics influencing explanation judgments, we do find that an expert who provides a better explanation is perceived as having more expertise.

### 5.2. Source Characteristics on Expertise

Our predictions about the importance of explanation satisfaction were well supported, but other hypotheses about source characteristics saw mixed results. Hypothesis 1 predicted that witnesses with more education would be evaluated as having more expertise, leading to more verdicts consistent with their testimony. Across the three studies, experts with higher education were consistently rated higher on general expertise, but this effect did not directly translate to verdicts. Similarly, Hypothesis 2 predicted that expert witnesses with more direct contact with the evidence would garner higher expertise ratings, leading to more verdicts consistent with their testimony. In Study 2, we did find that experts with more contact with the evidence received higher expertise ratings; however, this expertise was case-specific rather than general. In Study 1, experience significantly predicted verdicts, but this finding did not replicate in the later studies. Thus, for both source characteristics, it is not that they have no impact on any judgments, but we did not find consistent evidence that they impacted verdicts.

### 5.3. Expert Gender

The hypothesis that female experts would be rated less favorably and have less influence on verdicts received no support across any of the three studies. To the extent that we found gender differences, the female experts were rated more favorably, not less, than men—especially among experts with less advanced degrees. Though, as with the other source characteristics, gender differences were seen in our expertise ratings, but not in verdicts, and these effect sizes were relatively small.

This might appear to contradict findings in the previous literature that women with objectively higher expertise in an area are rated lower in expertise than men with objectively lower expertise (Thomas-Hunt & Phillips, 2004; Sonnentag & Volmer, 2009), as well as studies that demonstrate a bias against female expert witnesses (Larson & Brodsky, 2014; Maeder et al., 2012; McKimmie et al., 2004, 2013; Neal et al., 2012; Schuller et al., 2005), but this may reveal some promise regarding gender attitudes in STEM fields. It may be that seeing our female expert testifying about molecular biology might provide jurors with information that inadvertently counters negative gender stereotypes. It is also possible that a woman qualified as an expert by the court in such an impressive and male-dominated domain (like molecular biology) might have led mock jurors to engage in “re-fencing” (Allport, 1954), wherein they label them as exceptions to gender stereotypes, or “subtyping” (e.g., Weber & Crocker, 1983), wherein they put them in a separate subgroup—both of which allow people to maintain gender stereotypes without applying them in this specific circumstance. Alternatively, movements internationally that are focused on promoting women’s representation in STEM might have made mock jurors more aware of and motivated to correct for gender bias in this domain.

### 5.4. Expert Status Penalty and Explanation Satisfaction

We hypothesized that mock jurors might penalize those who they believe violate their expectations for an expert witness. Expert witnesses are likely presumed to be at a high level of expertise. Aligned with the shifting standards theory (Biernat & Manis, 1994), perhaps when a man has failed to achieve a higher degree (like a PhD), he violates expectations based on gender roles that set a standard of higher status. Knowledge about the difficulty for women to achieve high status in STEM fields, however, might prevent this backlash against female experts with a BA who are judged relative to traditional gender roles for women. Our findings might best support an explanation that men with less education receive a penalty, dragging down the overall average of male experts, not that women receive a boost for their gender—though these effect sizes are quite small and should be interpreted with caution as one data point in a larger span of literature.

Though when we look at this more broadly, we also did not find support for Hypothesis 5, that higher status experts would be penalized more for less satisfying explanations than would lower status experts, across source characteristics. We predicted that mock jurors would expect more from experts with a higher education, in particular, and would then penalize them more if they delivered a poor explanation—perhaps because, according to expectancy violations theory (Burgoon, 1993), they violate mock jurors’ expectations that an expert with a PhD, but not a BA, ought to deliver a satisfying explanation. However, what we found instead is that, independently, experts with a BA (versus a PhD) were perceived to have less expertise, and experts who gave a less (versus more) satisfying explanation were rated as having relatively lower expertise.

### 5.5. Limitations and Future Directions

As in any online jury experiment, we must be transparent as to how our study differs from a real-world context. Though research continues to bolster the credibility of online legal experiments (e.g., Irvine et al., 2018), online materials are shorter, less complex, and less vivid than an actual trial. In this study specifically, all the trial materials were presented via written text to participants. Despite work suggesting that written trial stimuli are not less valid than visual presentations (e.g., Bornstein, 1999; Maeder et al., 2024), written trial content may be less engaging or interactive to participants than if they were able to watch a trial and listen to an expert testify.

Another limitation is that the experts in all three studies testified about a single scientific technique. While we intentionally held the technique constant to control explanation content, it may be the case that source characteristics matter more for other kinds of evidence. We chose touch DNA as a relatively unfamiliar and complex type of evidence because past research found that reliance on source characteristics increases as scientific techniques become more complex (Cooper et al., 1996; Koehler et al., 2016), but perhaps other kinds of evidence would lead to a greater impact of source characteristics. For instance, perhaps listening to a forensic psychologist testifying about the mental state of a defendant could impact perceptions of expertise based on source characteristics (e.g., contact with the evidence—a forensic psychologist who personally interviewed a defendant may be seen as more expert than one who read an interview transcript). Further, given that other studies have found that the impact of expert gender depends on the domain, it is important for future research to continue to test whether these effects generalize across domains of expertise.

A third limitation relates to our explanation manipulation. While we replicated findings about the importance of explanation satisfaction on judgments across the three experiments, we tested a limited range of explanations and factors that could, presumably, affect explanation satisfaction. While the *less satisfying* explanation in Study 3 was rated as significantly less satisfying than the *more satisfying* explanation, it was still above the midpoint for mock jurors’ overall measured satisfaction. This is reassuring because it suggests that mock jurors still believed the witness was credible (even in the *less satisfying* condition), but we predict that further research might find an impact of source characteristics if the explanation was viewed as more unsatisfying or even objectively bad by mock jurors. Thus, our manipulation of explanation satisfaction (e.g., varying the quality, clarity, fluency) should be seen as a first step in uncovering what factors impact expert explanation satisfaction, and we encourage future research to assess different ways that an expert could provide jurors with a more (vs. less) satisfying explanation.

One (lack of) finding of note is that we did not find an effect of the expert’s education on verdicts. This is in contrast to some of the prior literature. We believe this may be due, at least in part, to the issue of the confounding of expertise and experience factors. Perhaps what was reported as the effect of education in past studies is really the effect of education and other implied characteristics that were tied to it, such as prestige, experience, or age. This in no way invalidates these prior findings: they found a real effect on verdicts. We propose, though, that these effects might not be the result of education alone but rather the schema associated with an advanced degree. Further studies could attempt to explore this possibility further.

Another avenue deserving of more study is the impact of an expert’s contact with the evidence. In Study 1, we found that an expert with direct contact with the case evidence had greater influence on verdicts than an expert more removed from the evidence. In Study 2, we found that experts with direct contact were also rated more highly on expertise factors having to do with the specific case in which they were testifying. While these findings were not as consistent as predicted, more research into different kinds of evidence contact (e.g., the expert collected versus processed the evidence, or the expert processed the evidence in this case, as well as in several related cases) could help better establish this effect. The fact that contact with the evidence did not relate directly to general expertise might also be due to the incorporation of a novel expertise scale. While the measure of expertise that we used consisted of theoretically face valid items and had good internal consistency (all Chronbach’s α > 0.85), it is important for future researchers to compare how this scale performs relative to validated scales on related constructs (e.g., expert witness credibility; Brodsky et al., 2010) in order to determine if this lack of findings is meaningful or due to measurement error.

One final direction that requires further exploration is our gender results. Our findings of a lack of bias against a female expert are surprising, given the context and stereotypes associated with women and expertise in this field. Perhaps our findings that women experts were seen as having more case-specific expertise than men were due to participants’ social desirability concerns to not appear sexist. However, because we did not have any measures of social desirability within this study, we cannot be sure and rule this out as an explanation. Further, we encourage caution about interpreting too much from these results because (1) while we did make sure to reinforce the gender of the expert throughout the trial and questions with pronouns and “Mr./Ms.”, we did not include a manipulation check for expert gender, and (2) the effects we did find had small effect sizes. Therefore, it is important that future research addresses these limitations by replicating these findings and extending them to different kinds of stimuli, different women, and different defendants. Because our photographs were all White women experts, we encourage exploration of intersectional effects in order to determine how favorably non-White women experts are rated relative to White women and White men.

## 6. Conclusions

Though attorneys may feel tasked to retain expert witnesses with the most impressive educational and experiential credentials, our findings provide one account to suggest that this may not be the best use of resources. Rather, attorneys would benefit from witnesses who can clearly explain their work and opinions to jurors. Across three studies, we examined the independent role of source characteristics related to expertise (i.e., education, contact with the evidence, expert gender) and explanation satisfaction on perceptions of the expert and verdicts. We found in this set of studies that it may not always be the qualities of the messenger but rather how the message is communicated that jurors rely on when evaluating expert testimony and deciding whether to use it in their verdict decisions. Consistently, mock jurors’ evaluations of explanation satisfaction predicted both perceptions of the expert’s expertise and overall verdict decisions. Given extensive evidence that source characteristics strongly influence jurors, future research should continue to examine when and how these traits affect juror decision-making. The present findings underscore that it is not just the expert’s qualifications, but also satisfaction with how their testimony is conveyed, that shapes juror judgments.

## Figures and Tables

**Table 1:** Means and standard deviations for general expertise ratings (Study 1).

Item	Study 1 *M* (*SD*)	Study 2 *M* (*SD*)	Study 3 *M* (*SD*)
How knowledgeable was [Dr./Mr.] Jones about touch DNA	6.33 (1.01)	6.46 (0.87)	6.24 (1.03)
[Dr./Mr.] Jones testimony helped me understand touch DNA	6.09 (1.13)	6.26 (0.98)	6.01 (1.14)
[Dr./Mr.] Jones is an expert on touch DNA	6.13 (1.06)	6.12 (1.10)	5.88 (1.21)
[Dr./Mr.] Jones is trustworthy	5.96 (1.14)	6.11 (1.04)	5.91 (1.10)
If I had a case where touch DNA was an issue, I would hire [Dr./Mr.] Jones to testify	5.69 (1.32)	5.79 (1.28)	5.56 (1.40)

Note. Items were rated on a 7-point Likert scale. Higher values indicate more positive evaluations of the expert.

**Table 2:** Mean (SD) expertise ratings as a function of education level across studies.

	Study 1		Study 2		Study 3	
	PhD	BA	PhD	BA	PhD	BA
General	**6.12 (0.91)**	**5.93 (0.99)**	**6.26 (0.78)**	**6.03 (0.88)**	**6.01 (0.92)**	**5.85 (1.02)**
Case-Specific	--	--	5.79 (0.99)	5.73 (1.07)	--	--

Note. Expertise measures were rated on 7-point scales. Bold indicates significant differences across conditions.

**Table 3:** Means and standard deviations for case-specific expertise ratings (Study 2).

Item	M	SD
How knowledgeable was [Dr./Mr./Ms.] Jones about the specific evidence in this case	5.87	1.12
[Dr./Mr./Ms.] Jones testimony helped me understand the specific evidence in this case	5.91	1.81
[Dr./Mr./Ms.] Jones is an expert on the specific evidence in this case	5.50	1.32

Note. Items were rated on 7-point Likert scales. Higher values indicate more positive evaluations of the expert.

## Data Availability

The data presented in this study are openly available in Open Science Framework at https://osf.io/t2dar/.

## Notes

1 We originally included a sixth item in the general expertise scale (“I relied on [Dr./Mr.] Jones testimony when I selected my verdict”); however, we removed this item from the scale post hoc because we believed it would artificially inflate the relationship between general expertise and verdicts.

2 Note, one participant reported their age as 369, which we suspect is a typo and omitted from the above age descriptives, but we maintained the rest of their data as it passed further checks.

## Supplementary Materials

The following supporting information can be downloaded at: https://bit.ly/4ryQvcf.
